# High physical activity is associated with post-traumatic stress disorder among individuals aged 15 years and older in South Africa

**DOI:** 10.4102/sajpsychiatry.v25i0.1329

**Published:** 2019-10-21

**Authors:** Karl Peltzer, Supa Pengpid

**Affiliations:** 1 HIV/AIDS/STIs and TB Research Programme, Human Sciences Research Council, Pretoria, South Africa; 2 Department of Research and Innovation, University of Limpopo, Turfloop, South Africa; 3 ASEAN Institute for Health Development, Mahidol University, Salaya, Thailand

**Keywords:** physical activity, post-traumatic stress symptoms, adolescents, adults, cross-sectional population survey, South Africa

## Abstract

**Background:**

Some research seems to suggest that physical activity (PA) was beneficial for post-traumatic stress disorder (PTSD).

**Aim:**

This study examined the association between levels of PA and PTSD among individuals 15 years and above in South Africa.

**Setting:**

Community-based survey sample representative of the national population in South Africa.

**Methods:**

In all, 15 201 individuals (mean age 36.9 years) responded to the cross-sectional South African National Health and Nutrition Examination Survey (SANHANES-1) in 2012.

**Results:**

One in five (20.1%) of participants reported exposure to at least one traumatic event in a lifetime, and 2.1% were classified as having a PTSD, 7.9% fulfilled PTSD re-experiencing criteria, 3.0% PTSD avoidance criteria and 4.3% PTSD hyperarousal criteria. Almost half (48.1%) of respondents had low PA, 17.4% moderate PA and 34.5% high PA. In logistic regression analysis, adjusted for age, sex, population group, employment status, residence status, number of trauma types, problem drinking, current tobacco use, sleep problems and depressive symptoms, high PA was associated with PTSD (odds ratio [OR] = 1.75, confidence interval [CI] = 1.11–2.75), PTSD re-experiencing symptom criteria (OR = 1.43, CI = 1.09–1.86) and PTSD avoidance symptom criteria (OR = 1.74, CI = 1.18–2.59), but high PA was not associated with PTSD hyperarousal symptom criteria. In generalised structural equation modelling, total trauma events had a positive direct and indirect effect on PTSD mediated by high PA, and high PA had a positive indirect effect on PTSD, mediated by psychological distress and problem drinking.

**Conclusion:**

After controlling for relevant covariates, high PA was associated with increased PTSD symptomatology.

## Introduction

Globally, the prevalence of post-traumatic stress disorder (PTSD) is significant and impacts morbidity and mortality.^1,2^ Compared with the general population, individuals with PTSD are more likely to have low physical activity (PA).^3^ In a systematic review from eight studies, four consistently found associations with lower PA in individuals with ‘PTSD symptoms of hyperarousal’.^3^ In additional studies, Whitworth et al.^4^ found that ‘strenuous intensity exercise’ directly decreased ‘avoidance/numbing and hyperarousal symptoms’, and total exercise directly decreased avoidance and numbing symptoms. LeardMann et al.^5^ found that engaging in PA, particularly high PA, decreased PTSD. All studies investigating levels of PA in relation to PTSD have been conducted in industrialised countries. In a previous review, Atwoli et al.^6^ note that ‘trauma and PTSD-risk factors may be distributed differently in lower-income countries compared with high-income countries’.

In several intervention studies, PA seems to be able to reduce symptoms of PTSD and depression among individuals with PTSD.^2,7^ Where limited access to traditional treatment modalities, such as psychotherapy and pharmacotherapy, of PTSD is available like in low-resourced settings such as in South Africa, PA intervention as an adjunct to PTSD treatment could be relevant.^2^ Based on prior studies,^3,8^ it was hypothesised that greater moderate and high PA levels would be associated with reduced overall PTSD symptoms and the three PTSD symptom clusters. The study aimed to examine the association between PA levels and PTSD among individuals aged 15 years and above in South Africa.

## Methods

### Sample and procedure

Cross-sectional data of the South African National Health and Nutrition Examination Survey (SANHANES-1) conducted in 2012 were analysed.^9^ Household members aged 15 years and above were ‘interviewed using a structured questionnaire on demographic and health variables’.^9^ The individual study survey response rate was 92.6%.^9^

### Measures

*Trauma event exposure.* Participants were asked, ‘Have you ever experienced any of the following events?’ (14 events, e.g. ‘severe automobile accidents’ and ‘learned about the sudden, unexpected death of a family member or a close friend?’ – Yes or No).^9^

*Post-traumatic stress disorder* was measured with the ‘17-item Davidson Trauma Scale (DTS)’ that assesses ‘all primary DSM-IV symptoms of PTSD related to intrusion, avoidance and hyperarousal symptoms’.^10^ Participants had PTSD ‘if they score at least one re-experiencing, three avoidance/numbing and two hyperarousal phenomena at a frequency of at least twice in the previous week’^10^ (Cronbach’s alpha 0.94).

*Physical activity* was assessed with the validated ‘General Physical Activity Questionnaire (GPAQ)’.^11,12^ ‘It assessed days and duration of PA at work, for transport, and during leisure time in a usual week’.^12^ Results were grouped into ‘low, moderate and high PA according to GPAQ guidelines’.^12^ Domain-specific PA (work, transportation and leisure time) were ‘classified into three groups, no (or low) activity, and low and high groups by the median metabolic equivalent (METs) of those having performed such activities’.^13^

*Sleep problems* was defined as ‘severe or extreme/can’t do’ having the ‘problem with sleeping, such as falling asleep, waking up frequently during the night, or waking up too early in the morning?’^14^

*Depressive symptoms* were defined as ‘severe or extreme/can’t do’ having the ‘problem with feeling sad, low or depressed’.^9^

Problem drinking was defined as scoring 3 or more in women and 4 or more in men on the Alcohol Use Disorders Identification Test–Consumption (AUDIT-C)^15^ (Cronbach’s alpha 0.89).

*Demographic data* included sex, age, population group, employment and residence status.

*Current tobacco use* included the use of ‘tobacco smoking and use of other tobacco products’.^9^

*Psychological distress* was defined as scores 20 or more on the 10-item Kessler scale^16^ that was validated in South Africa^17^ (Cronbach’s alpha 0.93).

*Body pains* were defined as ‘moderate, severe, extreme/can’t do’ having bodily discomfort.^9^

### Data analysis

Data analyses were conducted in STATA software version 15.0 (Stata Corporation, College Station, TX, USA), taking into account the complex study design. Multivariable logistic regression was used to estimate the effects of PA (and its domains) on PTSD (and PTSD symptom criteria), adjusted for age, sex, population group, employment status, residence status, number of trauma types, problem drinking, current tobacco use, sleep problems and depressive symptoms. Covariates were included based on the literature review.^3,4,5,8^ Possible two-way interactions were tested, but no significant indirect effects of low, moderate and high PA on PTSD symptoms or any of the individual PTSD symptom clusters (*p*s > 0.05) were detected. To investigate pathways of associations between (1) total trauma events and (2) high PA and PTSD, we built generalised structural equation models (GSEMs). Models included variables, such as bodily pain, sleep quality, psychological distress, alcohol consumption and substance use, that were previously used in assessing indirect effects of PA on PTSD.^4,18,19^ Maximum likelihood function with observed information matrix standard errors was used to fit the models using Akaike Information Criteria (AIC). Data that were missing were not included in the analysis and no collinearity was found.

### Ethical considerations

Informed written consent was obtained from participants. The study protocol was approved by the research ethics committee (REC) of the Human Sciences Research Council (REC 6/16/11/11).

## Results

### Sample characteristics

Participants included 15 201 individuals (of 16 780) 15–98 years (mean age 36.9, SD = 16.5) with complete measures of PTSD from South Africa. More than half of the participants were female (54.3%) and most were black African (77.8%), followed by white people (10.2%), mixed race (9.3%) and Indians or Asians (2.7%).

One in five respondents (20.1%) had exposure to at least one traumatic event in a lifetime based on DTS criteria. The specific major traumatic life events experienced included (1) ‘Sudden, unexpected death of a family member or a close friend’ (67.6%); (2) ‘Violent personal assault, serious accident, or serious injury experienced by a family member or a close friend’ (43.3%), (3) ‘Observed the serious injury or unnatural death of another person due to violent assault, accident, war, or disaster or unexpectedly witnessed a dead body or body parts’ (35.8%), (4) ‘Experienced severe automobile accidents’ (21.4%) and (5) ‘Being diagnosed with a life-threatening illness’ (16.7%). Regarding PTSD comorbidities, 2.9% of study participants reported ‘severe or extreme sleep problems’, 2.7% ‘severe or extreme depressive symptoms’, 20.5% engaged in problem drinking and 18.2% were currently using tobacco.

According to the three different PA levels, 48.1% of respondents were classified as having low PA, 17.4% moderate PA and 34.5% high PA. In all, 2.1% of study participants had a PTSD, 7.9% fulfilled PTSD re-experiencing criteria, 3.0% PTSD avoidance criteria and 4.3% PTSD hyperarousal criteria. Similar proportions of PTSD levels were found for the three PA domains (work, travel and leisure) (see Table 1).

**TABLE 1:** Sample characteristics.

Predictor variables (# missing cases)	Sample		PTSD (*n* = 299) (%)	PTSD re-experiencing (*n* = 998) (%)	PTSD avoidance (*n* = 414) (%)	PTSD hyperarousal (*n* = 558) (%)
	*N*	%				
All	15	201	2.1	7.9	3.0	4.3
**Age (# 10)**						
15–24	4296	27.7	2.0	6.8	2.6	3.7
25–44	5482	43.01	2.2	8.1	3.2	4.6
45–64	4015	21.9	2.3	8.6	3.2	4.5
65 or more	1398	7.3	2.1	8.6	3.3	4.5
**Sex (# 92)**						
Men	6292	45.7	2.0	8.6	3.3	4.9
Women	8817	54.3	2.3	7.0	2.8	3.7
**Population group (# 178)**						
Black African	10 045	77.8	2.5	8.5	3.5	4.7
White people	717	10.2	0.7	6.7	0.9	3.3
Mixed race	2983	9.3	0.9	3.8	1.5	1.7
Asian or Indian	1278	2.7	2.6	8.9	2.7	5.8
**Employment status (# 540)**						
No	9631	63.5	2.5	8.2	3.5	4.6
Yes	5030	36.5	1.6	7.4	2.2	3.9
**Residence (# 0)**						
Rural	5079	36.4	1.9	7.3	2.6	3.4
Urban	1022	63.6	2.3	8.2	3.3	4.9
**Number of trauma types (# 364)**						
0	12 186	79.9	0.0	0.0	0.0	0.0
1	978	7.4	6.1	28.9	8.4	11.7
2	695	5.0	10.1	35.2	14.9	20.1
3 or more	978	7.7	13.3	46.1	18.5	28.5
Sleep problems (# 103)	443	2.9	10.5	25.6	14.3	19.6
Depressive symptoms (# 105)	394	2.7	13.9	27.8	19.3	20.0
Problem drinking (# 311)	2816	20.5	3.5	10.0	4.6	6.1
Current tobacco use (# 344)	2990	18.2	3.4	10.2	4.5	6.2
**Physical activity (# 595)**						
Low	7278	48.1	1.7	6.7	2.4	3.9
Moderate	2504	17.4	1.8	7.6	2.9	3.7
High	4724	34.5	3.0	9.6	4.1	5.3
**Work physical activity (# 239)**						
Low (0 METS)	9770	62.8	1.6	6.5	2.3	3.5
Moderate (1–4789 METS)	2664	18.3	3.2	10.3	4.6	5.4
High (4800 or more METS)	2557	18.9	3.1	10.6	4.2	6.0
**Travel physical activity (# 239)**						
Low (0 METS)	6812	42.9	1.5	5.9	2.0	3.5
Moderate (1–799 METS)	3937	26.7	3.3	10.9	4.5	5.9
High (800 or more METS)	4436	30.4	2.1	8.2	3.2	4.2
**Leisure physical activity (# 362)**						
Low (0 METS)	12 395	79.3	2.0	7.1	2.8	3.9
Moderate (1–2039 METS)	1235	10.6	2.3	11.5	3.5	6.3
High (2040 or more METS)	1333	10.1	2.8	10.1	4.5	5.5

PTSD, post-traumatic stress disorder; METS, metabolic equivalents.

### Associations between physical activity levels and post-traumatic stress disorder

In logistic regression analysis, adjusted for age, sex, population group, employment status, residence status, number of trauma types, problem drinking, current tobacco use, sleep problems and depressive symptoms, high PA was associated with PTSD (odds ratio [OR] = 1.75, confidence interval [CI] = 1.11–2.75), PTSD re-experiencing symptom criteria (OR = 1.43, CI = 1.09–1.86) and PTSD avoidance symptom criteria (OR = 1.74, CI = 1.18–2.59), but high PA was not associated with PTSD hyperarousal symptom criteria (see Table 2).

**TABLE 2:** Associations between physical activity levels and PTSD (*N* = 13469).

Variable	OR	(95% CI)	*p*
**Criterion variable: PTSD**			
**Step 1**			
Low physical activity	1	Reference	-
Moderate physical activity	1.03	0.66–1.62	0.890
High physical activity	1.75	1.24–2.48	0.002
**Step 2**†			
Low physical activity	1	Reference	-
Moderate physical activity	1.43	0.88–2.34	0.148
High physical activity	1.85	1.18–2.89	0.008
**Criterion variable: PTSD re-experiencing symptoms**			
**Step 1**			
Low physical activity	1	Reference	-
Moderate physical activity	1.14	0.84–1.54	0.402
High physical activity	1.48	1.17–1.86	< 0.001
**Step 2**†			
Low physical activity	1	Reference	-
Moderate physical activity	1.34	0.96–1.85	0.082
High physical activity	1.46	1.11–1.90	0.006
**Criterion variable: PTSD avoidance and numbing symptoms**			
**Step 1**			
Low physical activity	1	Reference	-
Moderate physical activity	1.18	0.85–1.63	0.327
High physical activity	1.71	1.28–2.29	< 0.001
**Step 2**†			
Low physical activity	1	Reference	-
Moderate physical activity	1.60	1.03–2.48	0.036
High physical activity	1.90	1.28–2.82	< 0.001
**Criterion variable: PTSD hyperarousal symptoms**			
**Step 1**			
Low physical activity	1	Reference	-
Moderate physical activity	0.94	0.67–1.31	0.695
High physical activity	1.36	1.02–1.82	0.037
**Step 2**†			
Low physical activity	1	Reference	-
Moderate physical activity	1.17	0.78–1.76	0.440
High physical activity	1.46	0.99–2.15	0.054

OR, odds ratio; CI, confidence interval; PTSD, post-traumatic stress disorder.

† , Adjusted for age, gender, population group, residence status, employment status, number of trauma types, hazardous or harmful alcohol use, current tobacco use, sleep problems and depressive symptoms.

### Associations between domains of physical activity levels and post-traumatic stress disorder

In logistic regression analysis, adjusted for age, sex, population group, employment status, residence status, number of trauma types, problem drinking, current tobacco use, sleep problems and depressive symptoms, high work-related PA and moderate travel-related PA were associated with PTSD, while leisure-related PA was not associated with PTSD. High work- and leisure-related PA was positively associated with all three PTSD symptom criteria, while moderate and/or high travel-related PA was positively associated with all three PTSD symptom criteria (see Table 3).

**TABLE 3:** Associations between domains of physical activity levels and PTSD (*N* = 13469).

Variable	AOR	(95% CI)†	*p*
**Criterion variable: PTSD**			
**Work physical activity**			
Low (0 METS)	1	Reference	-
Moderate (1–4789 METS)	1.91	1.11-3.31	0.021
High (4800 or more METS)	1.83	1.08–3.11	0.026
**Travel physical activity**			
Low (0 METS)	1	Reference	-
Moderate (1–799 METS)	2.70	1.56–4.70	< 0.001
High (800 or more METS)	1.44	0.77–2.71	0.254
**Leisure physical activity**			
Low (0 METS)	1	Reference	
Moderate (1–2039 METS)	0.82	0.38–1.76	0.607
High (2040 or more METS)	1.35	0.76–2.41	0.311
**Criterion variable: PTSD re-experiencing symptoms**			
**Work physical activity**			
Low (0 METS)	1	Reference	-
Moderate (1–4789 METS)	1.44	1.09–1.90	0.010
High (4800 or more METS)	1.44	1.06–1.95	0.020
**Travel physical activity**			
Low (0 METS)	1	Reference	-
Moderate (1–799 METS)	2.40	1.71–3.36	< 0.001
High (800 or more METS)	1.52	1.08–2.15	0.017
**Leisure physical activity**			
Low (0 METS)	1	Reference	-
Moderate (1–2039 METS)	1.52	0.99–2.34	0.055
High (2040 or more METS)	1.46	1.02–2.08	0.039
**Criterion variable: PTSD avoidance and numbing symptoms**			
**Work physical activity**			
Low (0 METS)	1	Reference	-
Moderate (1–4789 METS)	2.07	1.39–3.08	< 0.001
High (4800 or more METS)	1.83	1.21–2.77	0.004
**Travel physical activity**			
Low (0 METS)	1	Reference	-
Moderate (1–799 METS)	2.99	1.82–4.91	< 0.001
High (800 or more METS)	1.65	0.95–2.85	0.073
**Leisure physical activity**			
Low (0 METS)	1	Reference	-
Moderate (1–2039 METS)	1.06	0.65–1.75	0.808
High (2040 or more METS)	1.84	1.12–3.03	0.017
**Criterion variable: PTSD hyperarousal symptoms**			
**Work physical activity**			
Low (0 METS)	1	Reference	-
Moderate (1–4789 METS)	1.51	1.04–2.19	0.031
High (4800 or more METS)	1.68	1.08. 2.60	0.022
**Travel physical activity**			
Low (0 METS)	1	Reference	-
Moderate (1–799 METS)	2.10	1.33–3.32	0.002
High (800 or more METS)	1.30	0.83–2.04	0.258
**Leisure physical activity**			
Low (0 METS)	1	Reference	-
Moderate (1–2039 METS)	1.31	0.74–2.34	0.353
High (2040 or more METS)	1.59	-	0.037

AOR, adjusted odds ratio; CI, confidence interval; PTSD, post-traumatic stress disorder; METS, metabolic equivalents.

† , Adjusted for age, gender, population group, residence status, employment status, number of trauma types, hazardous or harmful alcohol use, current tobacco use, sleep problems and depressive symptoms.

### Structural equation model analysis

**FIGURE 1:**
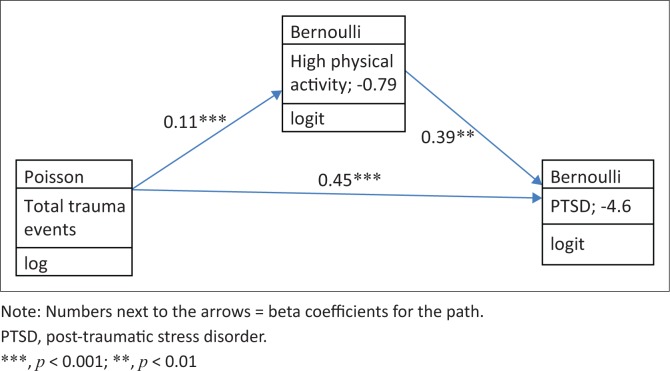
Generalised structural equation model legend: Total trauma events – high physical activity – post-traumatic stress disorder.

**FIGURE 2:**
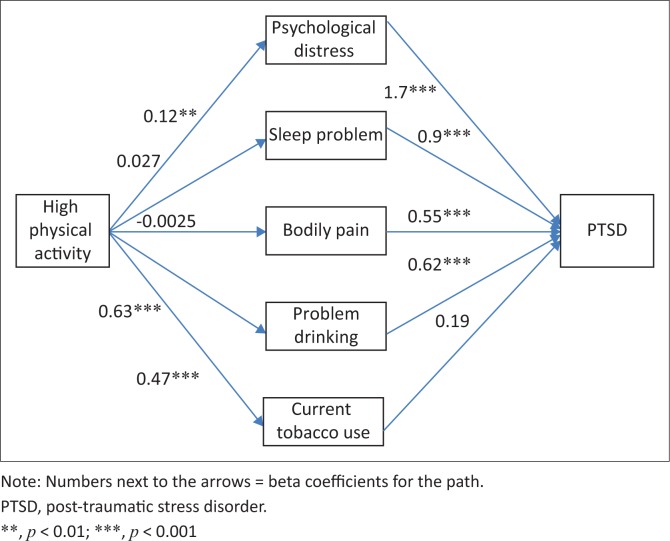
Generalised structural equation model legend: High physical activity – psychological distress (Kessler-10) – sleep problem – bodily pain – problem drinking – current tobacco use – post-traumatic stress disorder.

Total trauma events had a positive direct and indirect effect on PTSD mediated by high PA (see Figure 1). High PA had a positive indirect effect on PTSD, mediated by psychological distress and problem drinking (see Figure 2).

## Discussion

This is one of the first investigations in a middle-income country, South Africa, to assess the relationship between PA and PTSD. The current conditional prevalence of PTSD after trauma exposure found in this study was 2.1%, which seems a little lower than in the previous South African Stress and Health Study (3.5%).^20^ While in this study 20.1% reported at least experiencing one lifetime trauma, the previous study reported a much higher exposure of 73.8%.^20^ Differences may stem from the more comprehensive trauma exposure measure of the South African Stress and Health Study that used 27 different types of trauma exposure,^20^ while this study only had 14 different types of traumatic events.

This investigation found an association between high PA, after controlling for significant covariates, and PTSD total, PTSD re-experiencing and PTSD avoidance symptom criteria, but not with PTSD hyperarousal symptom criteria. These findings seem to be contrary to what previous studies found, namely associations between low PA participation and increased PTSD symptoms of hyperarousal,^3^ and high PA and decreased PTSD symptoms^5^ and decreased avoidance and numbing symptoms.^4^ One possible explanation for this difference could be that trauma and PTSD-risk factors as well as ameliorating factors such as PA may be distributed differently in low-income countries, such as South Africa, compared with high-income countries where the previous studies originated. Several studies^2,4,8^ found the beneficial effect of intensive exercise behaviour on PTSD and PTSD symptoms. However, when we analysed domain-specific PA, similar results were found that: in all three PA domains (work, travel and leisure-related PA) a positive association was found with PTSD and/or PTSD symptoms.

Although some other studies found an indirect effect of PA, for example via smoking^18^ and poor sleep quality,^19^ this study found positive indirect effects of PA on PTSD symptoms, mediated by psychological distress and problem drinking. Previous research^4,21,22^ has found that PA has beneficial effects on psychological distress and alcohol use, which was not supported by the findings of this study. However, several other studies^23,24^ found a positive relationship between PA and alcohol use. Furthermore, this study did not confirm previous findings,^18,19,25^ suggesting beneficial effects of PA on PTSD through tobacco use, sleep quality and bodily pain. Clearly, more longitudinal studies are needed to establish the direct and indirect links between PA activity and PTSD and PTSD symptoms.

### Study limitations

Because the investigation was based on cross-sectional data, no causative inferences can be made. The assessment of our data was based on self-report, including PA. This may have led to an overestimation of PA levels.^26^

## Conclusion

This investigation found in a national community-based sample in South Africa that, after controlling for relevant confounders, high PA was associated with overall PTSD symptoms, PTSD re-experiencing symptom criteria and PTSD avoidance symptom criteria. Future investigations in low- and middle-income countries are needed to replicate these results.
